# The molecular footprints of BK virus in the product of conception over the second and third gestational trimesters

**DOI:** 10.1186/s13104-023-06643-1

**Published:** 2023-12-11

**Authors:** Mona Shokoofeh, Somayeh Shatizadeh Malekshahi, Haleh Soltanghoraee

**Affiliations:** 1 Department of Virology, Faculty of Medical Sciences, Tarbiat Modares University, Tehran, Iran; 2 Reproductive Biotechnology Research Center, Avicenna Research Institute, ACECR, Tehran, Iran

**Keywords:** BK virus, Gestational trimester, Pregnancy, Nested-PCR, Vertical transmission

## Abstract

**Objective:**

Prior studies have shown controversial results on the vertical transmission of BK virus (BKV). The present study aimed to assess the possibility of BKV vertical transmission from mother to fetus in the product of conception (embryo, fetuses, and/or placentas) over the three stages of pregnancy.

**Results:**

Of the 26 placental studied tissues, 6 were in the first trimester, and none of which were positive. Only one out of the 13 (7.7%) placental materials in the second trimester was positive. Only one out of 7 (14%) placental materials of the third trimester was positive. There were cases that no virus was detected in their placental but BKV was detected in their other tissues. Among 26 conceptuses, 17 (65%) were negative for BKV and 9 (34.6%) were positive, 7/13 (54%) were positive in the second, and 2/7 (29%) were positive in the third trimester fetuses. BKV was most frequently detected in the liver (eight cases), heart (three cases), and placenta (2 cases). There were cases that no virus was detected in their placental but BKV was detected in their other tissues.

## Introduction

The BK virus (BKV) was first isolated from the urine of a Sudanese kidney transplant recipient by Gardner in 1971 [1]. BK viruses are small, non-enveloped, circular double-stranded DNA viruses belonging to the *polyomaviridae* family [2]. BKV isolates are classified into four subtypes (I, II, III, and IV) with unique geographic distribution. Subtype I is widespread all over the world with 80% frequency, followed by subtype IV with 15% frequency which is found in Europe and East Asia. In all geographic regions, subtypes II and III are rare (5%) [3]. The primary BKV infection is often subclinical and acquired early during childhood with more than 80% seroprevalence rate among adults [4]. Following the resolution of primary infection, the virus persists in the uroepithelium and renal tubular epithelial cells without complications for the immunocompetent host [5]. In the setting of immunosuppression, the virus undergoes reactivation and induces diverse clinical pathologies such as hemorrhagic cystitis, nephritis, encephalitis, retinitis, pneumonia, and ureteric stenosis [6]. The common mode of BKV transmission is presumed to be via a respiratory or a fecal-oral route but it may also be transmitted through semen, urine, organ transplantation, and blood transfusion [7, 8]. On the basis of immune system down regulation and hormonal changes during pregnancy, BKV may reactivate, and transplacental transmission might occur [9, 10]. Since transplacental transmission of polyomaviruses after inoculation of pregnant hamsters by SV40, [11] and mice by murine polyomavirus has been documented in animal models [12] it is conceivable that the same mode of transmission may occur in humans. Some authors have suggested the mother-fetus pathway as an alternative route of BKV transmission by detection of IgM in cord blood samples or BKV DNA in the placental and fetal tissues [13–16] but it is disputed and not confirmed by other researchers [17, 18]. Our study assessed whether BKV could be detected in embryos, fetuses, and/or placentas in the different trimesters, which would support vertical transmission from the mother.

## Materials and methods

### **Study populations**

This study utilized 58 formalin fixed paraffin embedded tissues (FFPE) from 26 fetopsies from women with unknown BKV serologic status. The tissue samples were selected from archives of the pathology lab of Avicenna Infertility Clinic, Tehran, Iran between 2017 and 2019. The histological slides from the first trimester were evaluated by an expert pathologist before laboratory analysis. Only those specimens containing embryo, fetal, or placental villus tissue were used, those containing maternal tissues or macerated tissue were excluded from the study. Ethical approval of the study was obtained from a medical ethics committee of Tarbiat Modares Univesity University (IR.MODARES.REC.1398.173).

### DNA extraction

**Fig. 1:**
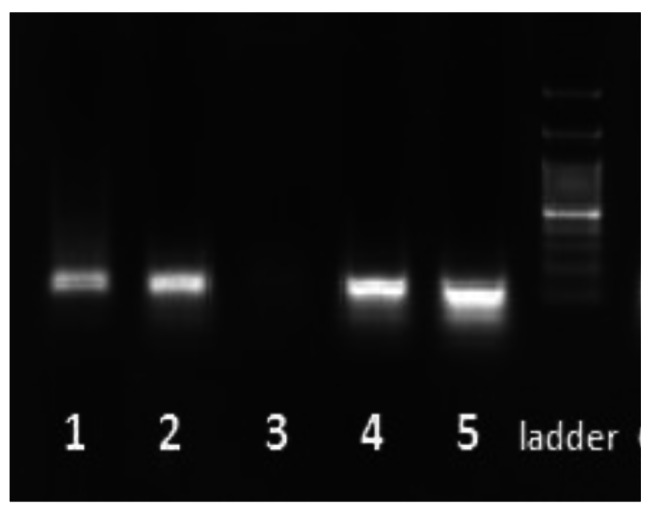
Agarose gel electrophoresis of beta-globin with a fragment length of 110 nucleotides

A 10-µm section was manually sectioned using disposable blades from the paraffin-embedded fetal and placental tissue samples and placed in a 1.5 ml sterile tube. Care was taken to avoid cross-contamination by frequent changing of gloves between samples. The blades were also changed before cutting the next block. The sections were deparaffinized with 1000 µl of xylene and treated twice with absolute ethanol to remove organic solvents. Then tissue samples were digested by lysis buffer (50 mM Tris-HCL pH 8.5, 1 mM EDTA, 150 mM NaCl, 1% SDS) containing 150 µl proteinase K at 37°C for overnight. Afterward, DNA was purified using phenol-chloroform method extraction followed by absolute cold ethanol precipitation and dissolved in TE buffer [1.1 gr Tris (PH = 8.1), d.d.H2O 1000ml, EDTA 0.37 gr]. The quantity was determined by a Nanodrop spectrophotometer at the end of the extraction procedure. The integrity of extracted DNA was evaluated by PCR using specific primers for the beta-globin gene which was amplified successfully in all 58 specimens (Fig. 1). The forward and reverse beta-globin primers were as follows: PC03: 5’-ACACAACTGTGTTCACTAGC-3’ and PC04: 5’-CAACTTCATCCACGTTCACC-3’ [19]. PCR amplification reaction was carried out in the 20 µl reaction mixture containing 10 µl Taq 2x Master mix RED-2 Mm Mgcl2 Ampliqon, 100–300 ng of target DNA, 0.5 µl (10 pmol) of each primer, 7 µl of double distilled water (D.D.W) with the following cycling conditions: 5 min initial denaturation step at 95 °C followed by 35 cycles of 95 °C for 20 s, 57 °C for 20 s and 72 °C for 30 s and a final 7-min elongation at 72 °C.

### Hemi nested PCR amplification

**Fig. 2:**
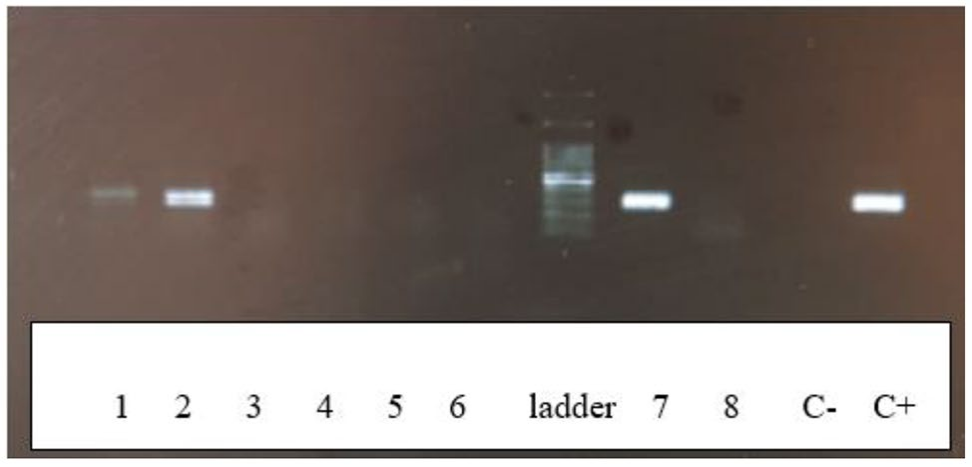
Agarose gel electrophoresis of hemi-nested PCR of BK virus with a fragment length of 302 nucleotides

BKV DNA detection in all FFPE samples (58 samples) was carried out by hemi nested PCR using BRP+/BRP2 primer pair (outer primers) and BRP1/BRP2 primer pair (inner primers) to obtain almost a 302 bp fragment of BKV transcriptional control region (TCR) gene. The sequences of primers were BRP + 5’-GAA AAAGCCTCCACACCC TTA-3’and BRP2 5’-GCCAAGATTCCTAGG CTCGC-3’ for the first round and BRP1 5’-ATGACACATTGGTGGTATATAG-3’ for the second round [14]. The hemi nested PCR reactions were performed in a 20 µL reaction mixture including 100–200 ng of DNA template, 10 µL of Taq 2x Master mix RED-2 Mm Mgcl2 Ampliqon, 10 pmol of each primer, and 7 DDW. PCR amplification cycles were as follows for the first and the second rounds: an initial 5-min denaturation at 95 °C, followed by 35 cycles of 95 °C for 20 s, 61 °C for 20 s, and 72 °C for 30 s, and a final elongation for 7 min at 72 °C. Double distilled water was used as the negative control in each set of PCR runs. A clinical specimen from previous studies with positive results for BKV was used as a positive control. The PCR products were run on a 1.5% agarose gel (Fig. 2).

## **Results**

### Clinical data and molecular analysis

The 26 pregnant women had a median age of 32 years [interquartile range (IQR), 28–35]. Table 1 lists the mother’s blood type, mode of pregnancy loss, and pregnancy type. Table 2 shows the 26 studied fetuses. For each one, the studied tissues, the mother’s age, gestational week, the trimester of pregnancy, and tissues that are positive for the presence of BKV have been listed. Six fetuses (with 6 FFPE tissues) with a mean age of 12 weeks were in the first trimester of pregnancy. Thirteen fetuses (with 29 FFPE tissues) with a mean age of 18 weeks were in the second trimester and 7 fetuses (with 22 FFPE tissues) with a mean age of 29 weeks were in the third trimester. Of the 26 placental studied tissues, 6 were in the first trimester, and none of which were positive. Only one out of the 13 (7.7%) placental materials in the second trimester was positive. Only one out of 7 (14%) placental materials of the third trimester was positive. There were cases that no virus was detected in their placental but BKV was detected in their other tissues. These included fetus no.16, positive for BKV in the heart, liver, and thymus during the second trimester but was not detected in the placenta, and fetus no. 21 BKV was detected in the heart tissue in the third trimester. In fetuses no. 19, 23, 24, 25, and 26 the BKV was detected only in liver tissue and all of them were in the second trimester.

**Table 1:** Clinical data of the 26 pregnant women with aborted fetuses

Mode of pregnancy loss	No. (percent)	Pregnancy type	No. (percent)	Couple relation	No. (percent)	Blood group	No. (percent)
Cesarean	1 (4.2%)	IVF	2 (9.1%)	No relationship	16 (73%)	A^+^	5 (22%)
Spontaneous abortion	15 (62%)	Physiological	19 (86%)	Has relationship	6 (27%)	A^-^	1 (4.3%)
Drug-induced	8 (33%)	By drug usage	1 (4.5%)	Undetermined	4	B^+^	3 (30%)
Undetermined	2	Undetermined	4	**-**	**-**	O^+^	7 (7%)
Undetermined	**-**	Undetermined	**-**	Undetermined	**-**	O^-^	3 (13%)

**Table 2:** The 26 studied fetuses and their related data

Case	Pregnancy trimester	Tissues obtained from each fetus	Mother’s age (year)	Gestational age (week)	BK virus PCR result
1	First	Placenta	29	12	Negative
2	First	Placenta	37	10	Negative
3	First	Placenta	28	12	Negative
4	First	Placenta	N/A	12	Negative
5	First	Placenta	31	12	Negative
6	First	Placenta	34	12	Negative
7	Second	Placenta	32	18	Negative
8	Second	Placenta	34	21	Negative
9	Second	Placenta	39	27	Negative
10	Second	Placenta	43	17	Negative
11	Second	Placenta	29	23	Negative
12	Third	Placenta/Liver/Heart	28	28	Negative
13	Third	Placenta/Liver/Heart	34	40	Negative
14	Third	Placenta/Liver/Heart/Thymus	28	29	Positive in Placenta/Liver/Heart
15	Third	Placenta/Liver/Heart/Kidney	35	31	Negative
16	Second	Placenta/Liver/Heart/Thymus	38	15	Positive in Liver/Heart/Thymus
17	Second	Placenta/Liver	40	17	Positive in Placenta/Liver
18	Second	Placenta/Liver/Heart	36	16	Negative
19	Second	Placenta/Liver/Heart		15	Positive in liver
20	Third	Placenta/Liver/Heart/Gonad	40	28	Negative
21	Third	Placenta/Heart	29	39	Positive in Heart
22	Third	Placenta/Heart	28	28	Negative
23	Second	Placenta/Liver/Heart	25	21	Positive in liver
24	Second	Placenta/Liver/Heart	27	26	Positive in liver
25	Second	Placenta/Liver/Heart	35	21	Positive in liver
26	Second	Placenta/Liver/Heart	18	19	Positive in liver

N/A: Not available

Of these 26 fetuses, 17 (65%) were negative for BKV and 9 (34.6%) were positive. Of 9 positive fetuses, 7/13 (54%) were in the second trimester of pregnancy and 2/7 fetuses (29%) were in the third trimester. BK virus was not detected in any of the 6 fetuses in the first trimester.

## Discussion

Numerous studies have shown horizontal transmission of BKV through various routes such as fecal-oral or respiratory [20]. However, limited studies have been performed on mother-to-child transmission of *Polyomaviruses* and there is little information about the vertical transmission of BKV. In this study, 57 FFPE tissues from 26 fetuses in the first, second, and third trimesters of pregnancy were examined for the presence of the BKV DNA by hemi nested PCR. Among them, 17 (65%) were negative and 9 (34.6%) were positive for the presence of BKV. Of these 9 positive fetuses, 7 fetuses (54%) were in the second trimester of pregnancy and 2 fetuses (29%) were in the third trimester. BK virus was not detected in any of the 6 embryos in the first trimester. BKV genome was detected most frequently in the liver (9/26 case) and heart (3/26 case) and in four cases, BKV infection involved two or more organs (Table 2).

Prior studies have shown controversial results on the vertical transmission of BKV. Some groups have reported the presence of BKV in mother and fetus tissues although this has been denied by others. Pietropaolo V et al. investigated the BKV DNA in autopsy specimens (placenta, brain, and kidney) of 15 aborted fetuses and maternal tissues during 20–24 weeks of pregnancy. Nine cases showed the presence of BKV in all organs tested and three were positive in only the placenta and brain. Their results suggested that due to the simultaneous presence of the BKV genome in maternal and fetal materials vertical transmission may be the predominant mode of BKV transmission [10]. Boldorini R et al., investigated blood and urine samples from 300 pregnant women in the third trimester, as well as umbilical cord blood samples of their newborns. BKV DNA was detected in 28 (9.3%) of the urine and 10 (3.3%) of the maternal blood samples but in none of the umbilical cord blood samples. We also detected BKV sequences in a limited number of placenta tissues (only 2 of the 26 placental tissues) (Table 2). The authors concluded that transplacental crossing of BKV in the offspring is not a significant route of BKV transmission [21]. It could be assumed that transmission of the BKV from mother to fetus is possible via both placental and ascending routes, and this issue requires further study. The same group investigated BKV DNA sequences in various organs sampled from 10 aborted fetuses. Then, the virus was detected in 7 out of 10 aborted fetuses most frequently in heart and lung (five cases). The placenta was negative in all but one case [14]. It is in line with our study in terms of low virus detection in placental tissue while other obtained tissues of the fetuses were positive.

Based on the results of a previous study of autopsied kidneys, the BKV genome was detected only in some of the specimens taken from the same renal tissue, suggesting that viral DNA is focally distributed [22]. In our study, it may also be hypothesized that one reason for the absence of the BKV sequences in some tissues of the placenta and other organs in the fetuses with one or more BK-positive tissues is that the virus is focally distributed in tissue. If samples are taken from different parts of the placenta, evidence of the virus may also be found in those tissues.

In the study by Kalvatchev Z et al., BKV DNA was detected in 18 of 52 pregnant women’s urine (34.6%) and none of 51 cord blood samples. The authors concluded that BKV reactivation and urine excretion occur during pregnancy but this is not associated with BKV in cord blood [13]. In a recent study, BKV vertical transmission was revealed by the presence of BKV DNA sequences and IgG against BKV in umbilical cord blood (UCB) samples [15].

It should be noted that different observed prevalence of BKV DNA sequences reported in distinct studies could be due to different techniques used, investigated populations, and study duration.

## **Conclusion**

This study conducted in Iran investigated the transmission of BKV from mother to fetus. According to the data obtained in this study, BKV transmission from mother to fetus during the second and third trimesters of pregnancy is possible. BKV was detected in the liver tissue more frequently than in the heart and placenta samples. In this respect, it can be hypothesized that transmission of the BKV from mother to fetus is possible through both placental and ascending modes and this issue needs further studies by examination of BKV in urine and blood samples of pregnant women to evaluate the likelihood of active BKV infection.

### Limitations

The study’s limitation was its small sample size. Some pregnant women were excluded due to incomplete data in their medical records.

## Data Availability

The data that support the findings of this study are available from the corresponding author upon reasonable request.

## List of abbreviations

BKV: BK virus
FFPE: Formalin fixed paraffin embedded tissues
TCR: Transcriptional control region
IQR: Interquartile range
UBC: Umbilical cord blood
